# The seesaw effect of winter temperature change on the recruitment of cotton bollworms *Helicoverpa armigera* through mismatched phenology

**DOI:** 10.1002/ece3.1829

**Published:** 2015-11-17

**Authors:** Gadi V. P. Reddy, Peijian Shi, Cang Hui, Xiaofei Cheng, Fang Ouyang, Feng Ge

**Affiliations:** ^1^ Western Triangle Agricultural Research CentreMontana State University9546 Old Shelby RoadP.O. Box 656ConradMontana59425, USA; ^2^ Collaborative Innovation Center of Sustainable Forestry in Southern China of Jiangsu ProvinceNanjing Forestry University159 Longpan RoadXuanwu DistrictNanjing210037China; ^3^ Department of Mathematical SciencesCentre for Invasion BiologyStellenbosch UniversityMatieland7602South Africa; ^4^ Mathematical and Physical BiosciencesAfrican Institute for Mathematical SciencesCape Town7945South Africa; ^5^ State Key Laboratory of Integrated Management of Pest Insects and RodentsInstitute of ZoologyChinese Academy of Sciences1 Beichen West RoadChaoyang DistrictBeijing100101China

**Keywords:** Global warming, *Helicoverpa armigera*, mismatched phenology, pest outbreaks, seesaw effect

## Abstract

Knowing how climate change affects the population dynamics of insect pests is critical for the future of integrated pest management. Rising winter temperatures from global warming can drive increases in outbreaks of some agricultural pests. In contrast, here we propose an alternative hypothesis that both extremely cold and warm winters can mismatch the timing between the eclosion of overwintering pests and the flowering of key host plants. As host plants normally need higher effective cumulative temperatures for flowering than insects need for eclosion, changes in flowering time will be less dramatic than changes in eclosion time, leading to a mismatch of phenology on either side of the optimal winter temperature. We term this the “seesaw effect.” Using a long‐term dataset of the Old World cotton bollworm *Helicoverpa armigera* (Hübner) (Lepidoptera: Noctuidae) in northern China, we tested this seesaw hypothesis by running a generalized additive model for the effects of the third generation moth in the preceding year, the winter air temperature, the number of winter days below a critical temperature and cumulative precipitation during winter on the demography of the overwintering moth. Results confirmed the existence of the seesaw effect of winter temperature change on overwintering populations. Pest management should therefore consider the indirect effect of changing crop phenology (whether due to greenhouse cultivation or to climate change) on pest outbreaks. As arthropods from mid‐ and high latitudes are actually living in a cooler thermal environment than their physiological optimum in contrast to species from lower latitudes, the effects of rising winter temperatures on the population dynamics of arthropods in the different latitudinal zones should be considered separately. The seesaw effect makes it more difficult to predict the average long‐term population dynamics of insect pests at high latitudes due to the potential sharp changes in annual growth rates from fluctuating minimum winter temperatures.

## Introduction

Knowing how climate affects the dynamics of agricultural insects is important to maintain ecosystem services and food security (Tian et al. 2011; Weed et al. 2013). Compared to tropical insects, insects from higher latitudes have a broader thermal performance range and often experience climates below their physiological optimum, and a warming climate is likely to increase their fitness and the magnitude of their outbreaks (Deutsch et al. 2008). One crucial aspect of climate change is the change in temperature extremes. The poleward distribution of insects in the northern hemisphere, such as the southern pine beetle (*Dendroctonus frontalis* Zimmermann (Coleoptera: Curculionidae) and the yellow stem borer (*Scirpophaga incertulas* Walker (Lepidoptera: Crambidae), is often limited by the minimum winter temperature (MWT hereafter) (Uvarov 1931; Ungerer et al. 1999; Shi et al. 2012a), probably through affects on development and population growth of these species (Tran et al. 2007; Friedenberg et al. 2008; Shi et al. 2012b). While increasing MWT can sometimes benefit the population growth of *D. frontalis* (Tran et al. 2007; Friedenberg et al. 2008), *S. incertulas* populations decline in response to rising MWT (Shi et al. 2012b). Such disparity in how insects respond to rising winter temperatures warrants careful investigation.

**Figure 1:**
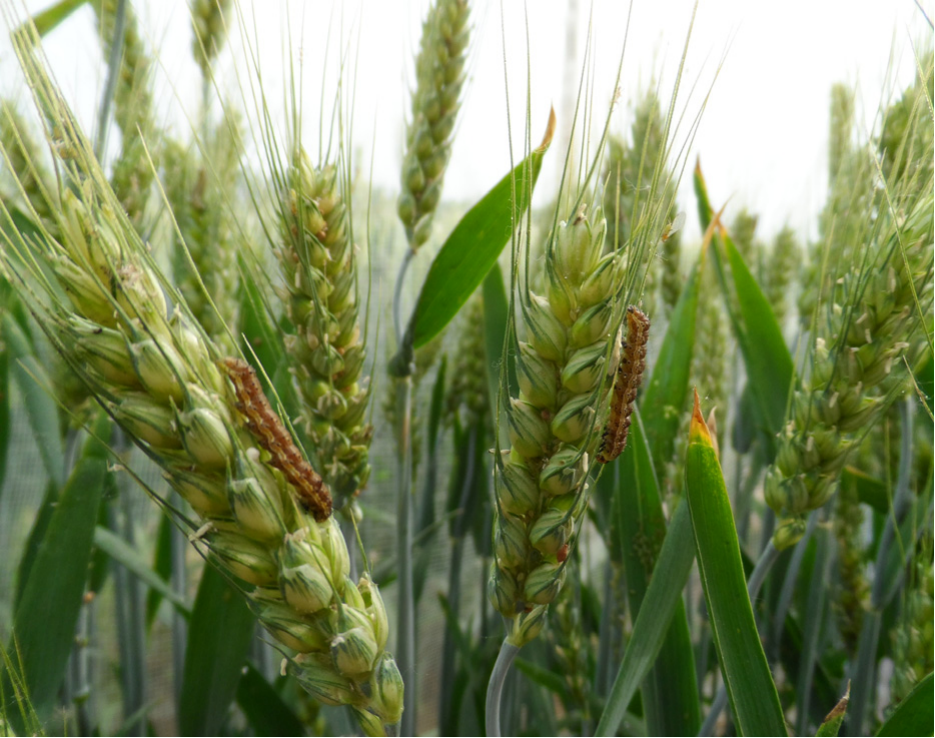
The first generation larvae of *Helicoverpa armigera* feeding on wheat.

The Old World cotton bollworm *Helicoverpa armigera* Hübner (Lepidoptera: Noctuidae) is a widely distributed agricultural pest around the globe, causing damage to many important crop species (Zhang and Zhao 1996). In northeast Asia, it undergoes four generations of moths per year, with adults of the overwintering generation that emerge from diapausing pupae feeding on nectar from flowers of wheat, the first generation larvae feeding on wheat (Fig. 1) and the other three generation larvae feeding on cotton and corn. The fourth generation larvae further develop to pupae that then enter a state of diapause in autumn (Ge et al. 2005). Winter air temperature, especially minimum temperature, can affect the survival rate of overwintering insect populations. While increased MWT might enhance the survival of diapausing insects (Ungerer et al. 1999; Shi et al. 2012a), unusually warm winter temperatures might retard the growth rate of cotton the following spring. From 1951 to 1990, MWT across northern China increased by 0.5–0.7°C per decade (Zhai and Ren 1997). In addition, the urban heat island effect due to urbanization over the northern China has also increased the magnitude and variation of the extreme minimum temperatures in and around cities (Li et al. 2014). During the recent 20 years, the population of cotton bollworms in northern China is reported to few exhibit local outbreaks, and it is believe to be attributable to the wide plantation of the transgenic crops, especially the transgenic cotton that used the crystal protein endotoxin genes from the bacterium *Bacillus thuringiensis* (Bt) (Wu et al. 2008). However, Ouyang et al. (2014) debated that the climate change and agricultural intensification change also might lead to the great fluctuation of population abundances of cotton bollworm. In this case, it is critical to study how the cotton bollworms, especially the overwintering generation, respond to such rapidly rising MWT.

Although the survival rate of overwintering *H. armigera* pupae can increase when exposed to increasing winter air temperatures, adult moths in spring might face a scarcity of food. This is because of wheat flowering likely has a different response than the pupae to rising winter temperatures. In general, plant development is more strongly influenced by effective accumulated temperatures than is the development or emergence of multivoltine insects. Thus, rising winter air temperature might result in a timing mismatch between wheat flowering and bollworm moth eclosion. If this is so, we would expect to see a seesaw effect of rising winter temperature on the population dynamics of overwintering generation cotton bollworms. The seesaw effect means that only the temperate winter temperature can benefit the population increase of cotton bollworms and extremely low or extremely high winter temperatures will do harm to the population of cotton bollworm. This effect is mainly attributable to the mismatch between the flowering peak of wheat and the eclosion peak of cotton bollworms overwintering generation. There are only wheat as host plants when the overwintering generation moths occur. And at the same time, the cotton seeds are only sowed, so the cotton bollworm moths and even its first generation larvae cannot feed on cotton.

In this study, we attempted to explore: (1) whether the abundance of the third generation moth of bollworms can influence the abundance of the overwintering generation in the following year; (2) which climatic factors may significantly affect the population dynamics of the overwintering generation of cotton bollworms; and (3) whether there is a seesaw effect due to variation in the minimum winter temperature on population dynamics of cotton bollworm. In addition, we sought to provide a framework for analysis for detection of the seesaw effect of matched or mismatched insect/plant phenology.

## Materials and Methods

In the east plain of northern China (i.e., Huabei Plain), there are usually four generations of cotton bollworm moths: the overwintering generation moth and the first to the third‐generation moths. Because the developmental time of female immature stage (egg, larva, and pupa) is larger than the life span of female adults (5.2 times at 15°C, 2.5 times at 20°C and 25°C, and 1.8 times at 30°C; Wu et al. 1980), there are few moth overlapping between two adjacent generations. However, it frequently occurs between the moths of the previous generation and the eggs, larvae even pupae of next generation in the field. Thus, we can find an obvious break of moth number trapped by lights between the adjacent generations. It is easy to distinguish the third‐generation moths from the second generation moths. For the next overwintering generation, it is easier to distinguish them from the third generation of preceding year because of a long winter.

**Figure 2:**
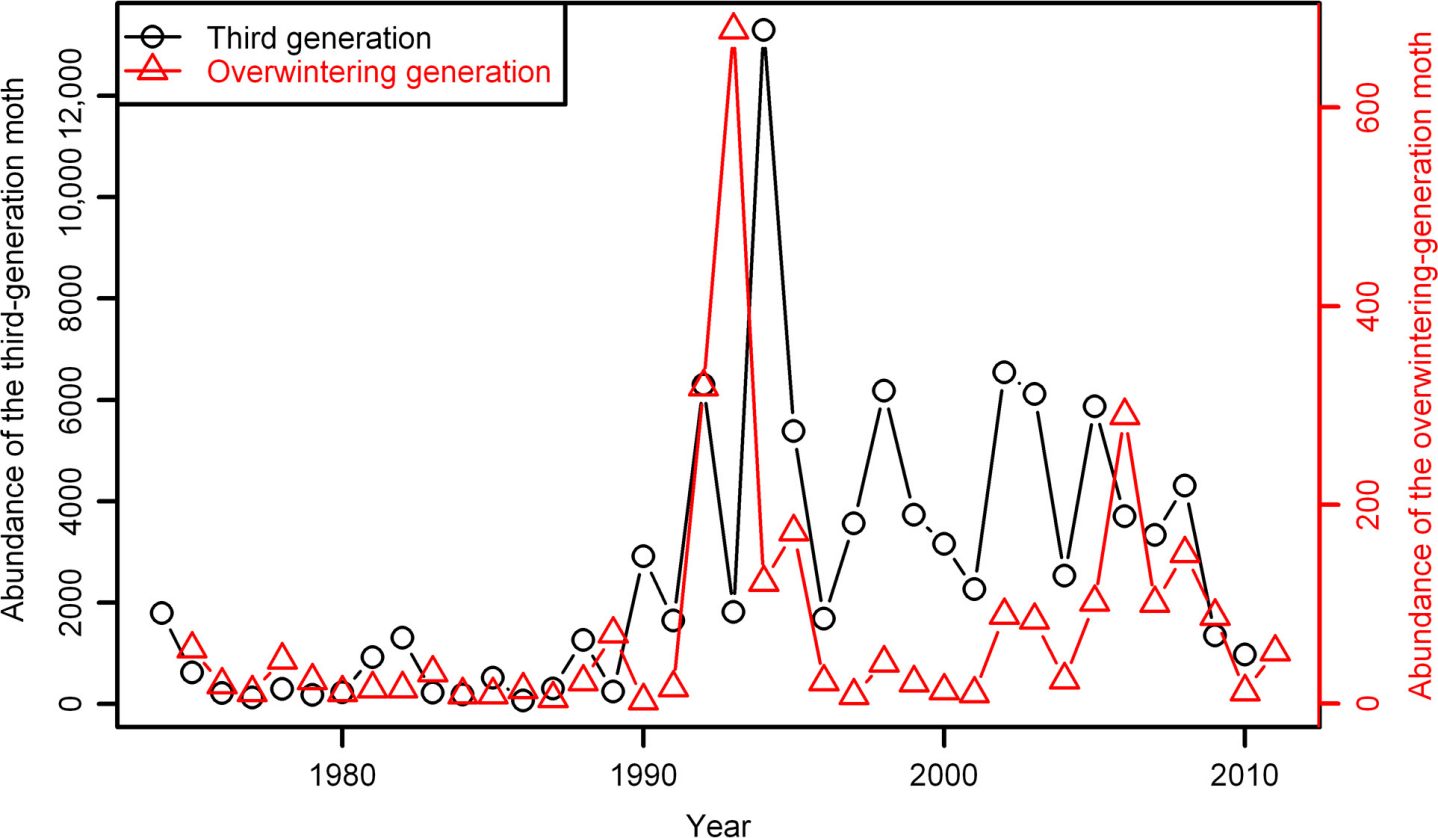
Comparison of population dynamics of the third generation moth of *Helicoverpa armigera* of the preceding year and the overwintering generation moth in Raoyao County, in northern China.

The abundances of the overwintering generation and the third generation of cotton bollworm were compared from 1975 to 2011 using a light trap at a site (38°14′11′′N, 115°42′32′′E) located in White Pond Village, Raoyang County of Hebei Province, China (Fig. 2). Details of the landscape surrounding the monitoring site can be found in Ouyang et al. (2014). Data on the MWTs, monthly precipitation, and monthly average temperatures in winter from the same period were acquired from the China Meteorological Data Sharing Service System (cdc.nmic.cn). We then calculated the winter average temperature (WAT) using the day‐number weighted average of monthly average temperature of January, February, and December of the preceding year, as well as the accumulated winter precipitation of the 3 months.

**Figure 3:**
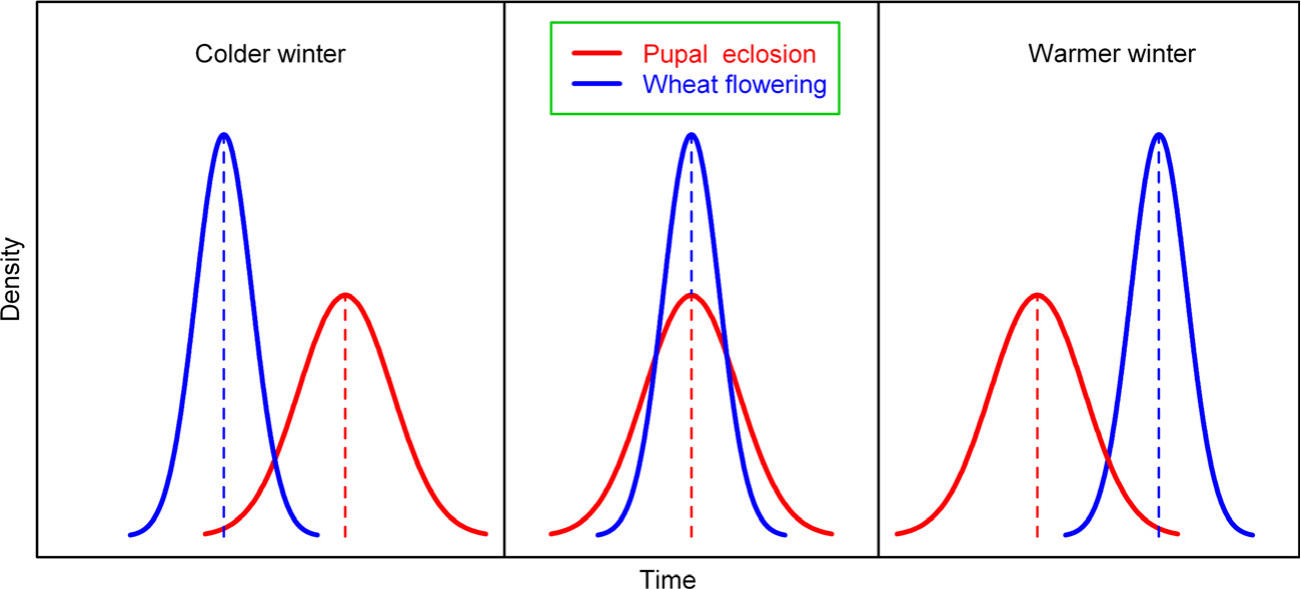
Conceptual diagram of the hypothesis of phenological mismatch between wheat flowering and pupal eclosion as driven by extreme cold or warm winter temperatures.

Insects have smaller body size and higher minimum developmental thresholds (or called “lower developmental threshold” (LDT)) than most plants. The LDTs of most insects, mites, and spiders are on average, 10°C (Li and Jackson 1996; Bonato et al. 2011; Kiritani 2011; Miller 2011), although aphids have lower LDTs, with a mean level of just 5°C (Bonato et al. 2011). However, the LDTs of flowering plants are generally are lower, being near 0°C (e.g., Leopold 1964; Aono 1993; Diekmann 1996). Plants have a lower developmental threshold so that they can grow during cool periods when air temperatures have not reached the developmental threshold of pests such as cotton bollworms. One previous study reported that the developmental threshold of cotton bollworms could exceed 12°C (Ikemoto and Egami 2013). With rising winter temperatures, there might be a difference in the timing of important events in plant vs. insect life histories (Fig. 3). In northern China, the abundance of moths of the first generation of cotton bollworms depends on whether the peak emergence of moths from overwintering pupae correctly matches the timing of flowering of wheat, as wheat nectar is a key resource for adult cotton bollworms in this period. Access to adequate nectar sources is needed to provide moths with ample carbohydrates for the production of a large number of high quality eggs. Such a match can only be achieved with a moderate MWT (Fig. 3); extremely cold and warm winters both lead to an important mismatch of insect and plant phenology. We call this fine balance of plant and insect phenology the seesaw effect of rising winter temperature.

We explored the effects of the following variables on the effective abundance of overwintering cotton bollworm adults: (1) the abundance of the third‐generation moths in the preceding year; (2) MWT (also replaced by the average winter temperature in a separate analysis); and (3) the number of winter days with the lowest daily temperature below −12°C. Generalized additive models (GAMs) have been widely used for exploring complex biological interactions (e.g., Xu et al. 2011; Shi et al. 2012b; Yan et al. 2013). Considering the possible nonlinear effects of these predictors, we used the GAM to fit the data (Hastie and Tibshirani 1990; Wood 2006). We then conducted a backward model selection by dropping one insignificant variable and then rerunning the GAM for reduced sets of parameters. We used a linear regression of MWT values from 1957 to 2012 to test whether winter temperatures showed an overall rising trend.

We chose −12°C as a critical temperature for the following reason. An insect can quickly die when exposed to temperatures at or below its supercooling or freezing point. There is a significant difference in the supercooling point for diapausing pupae feeding on Bt cotton vs. non‐Bt cotton, −16.8°C vs. −19.5°C (Ouyang et al. 2011). Previous studies have shown that the supercooling point of cotton bollworms in the study area ranged from −20 to −19°C, and the freezing point from −10 to −8°C (Wu et al. 1997; Yang et al. 2003). Because diapausing pupae generally overwinter 3–5 cm beneath soil surface (Chen et al. 2002), they experience soil temperatures that are 2–4°C warmer than air temperature (Yang 2014). To further examine the effect of the chosen critical temperature (−12°C), we used a range of temperature, from −14 to 0°C, in increments of 0.1°C. We then reran the GAM of the abundance of moths of the overwintering generation on (1) the abundance of moths of the third generation of the previous year; (2) the MWT; and (3) the number of winter days below the candidate critical temperatures. The goodness of fit using different candidate critical temperatures was then compared.

## Results

**Figure 4:**
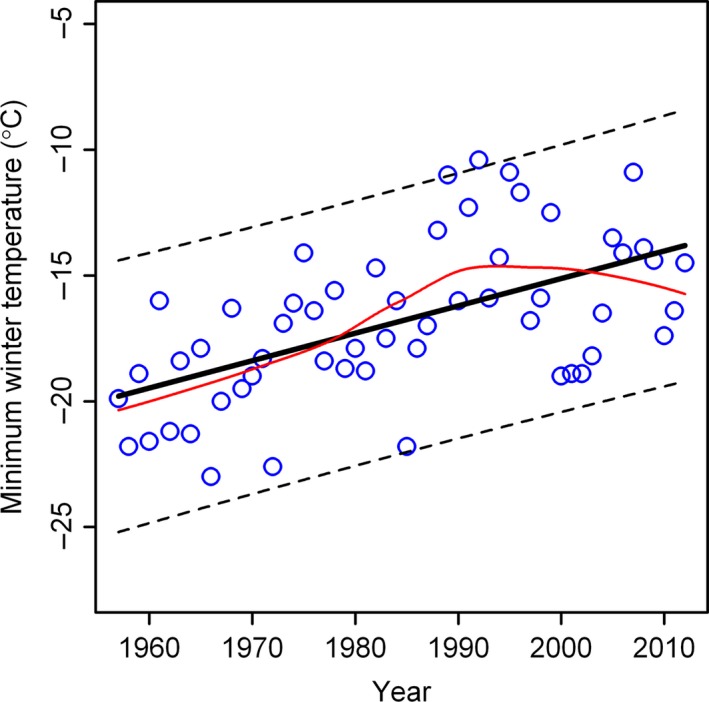
Trends in minimum winter temperature over time in Raoyang County in northern China, where small open circles represent observed MWT, the bold straight line represents the fitted values of MWT based on the linear regression, and the red curve represents the predicted values of MWT based on the local regression.

The MWT in Raoyao has increased significantly over the last 56 years (Fig. 4), with an annual increment of 0.11°C (95% confidence interval: [0.066, 0.152], *P *<* *0.01), and an overall increase of 6.2°C in the MWT. The MWT was significantly correlated with the average winter temperature (*r *=* *0.45, *P *<* *0.01).

**Figure 5:**
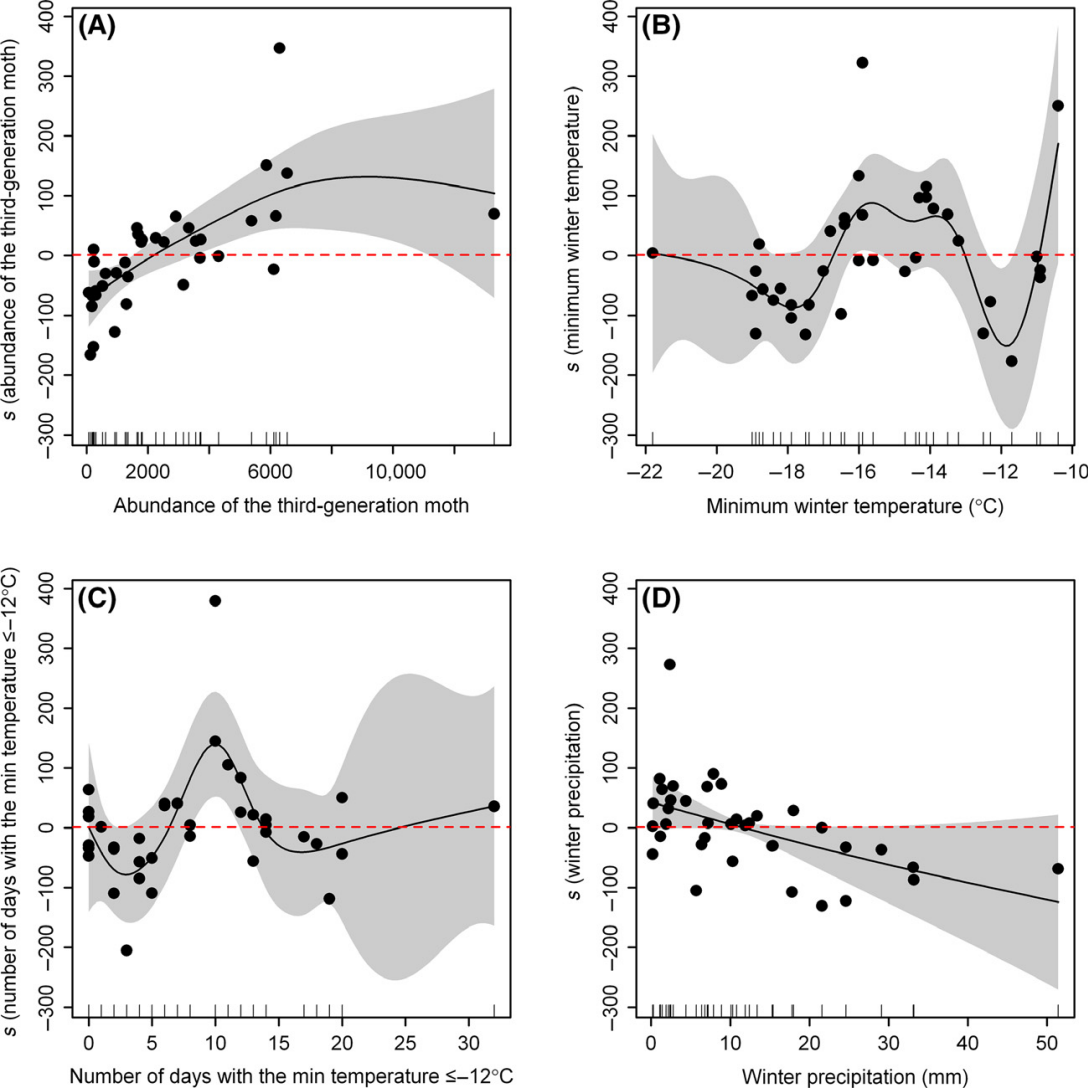
Generalized additive model predictions of the abundance of the overwintering generation of *Helicoverpa armigera* based on four variables. (A) Smooth and partial residuals of abundance of the third generation moth from the preceding year; (B) smooth and partial residuals of minimum winter temperature; (C) smooth and partial residuals of number of days with the minimum air temperature ≤−12°C in winter; (D) smooth and partial residuals of the accumulated precipitation in winter.

Of the four variables, two (number of days with the daily minimum temperature ≤−12°C and accumulated winter precipitation) were found to be insignificant, while the other two variables (the abundance of adult cotton bollworm moths in the third generation of the preceding year and the MWT) had significant effects on the abundance of the overwintering generation (Table 1; Fig. 5). Both the MWT and the number of winter days with the daily minimum temperature at or below −12°C had a seesaw effect on the abundance of the overwintering generation moth (Fig. 5B and C).

**Table 1:** Generalized additive model fit to the abundance of the overwintering generation of *Helicoverpa armigera* using four predictive variables

Item	df	*F*	*P*	$R_{\mathrm{adj}}^{2}$	Variance explained
s(*x*_1_)	2.444	5.421	0.010	0.543	75%
s(*x*_2_)	8.010	3.130	0.018		
s(*x*_3_)	7.019	1.917	0.120		
s(*x*_4_)	1.226	2.709	0.107		

Here, s represents the smooth function, *x*_1_ represents the abundance of the third generation of adult cotton bollworms of the last generation of the preceding year, *x*_2_ represents minimum winter temperature, *x*_3_ represents the number of days with the minimum daily air temperature ≤−12°C in winter, and *x*_4_ represents the accumulated precipitation in winter.

Although the MWT and the number of winter days with a daily minimum below −12°C are negatively correlated with each other (*r *=* *−0.63, *P *<* *0.01), removing either one sharply reduced the goodness of fit of the model to the data. These two variables were thus kept in the model. As the accumulated winter precipitation was found to be insignificant, we dropped the variable during model development. Interestingly, a model with only the three remaining variables had a better goodness of fit (Table 2), raising the adjusted *R* ^2^ from 0.543 to 0.689. The effects of all three variables were found to be significant, as in the full model. MWTs in the range of −17 to −13°C were found to be beneficial to the overwintering pupae; on either side of this range, MWTs reduced moth population growth. A similar seesaw effect was found for the variable “number of days with the minimum daily temperature ≤−12°C,” for which the optimal range was 7 to 14 days, while either greater or lower values had a negative effect on moth density.

**Table 2:** Generalized additive model fit to the abundance of the overwintering generation of *Helicoverpa armigera*, using three predictive variables, including minimum winter temperature

Item	df	*F*	*P*	$R_{\mathrm{adj}}^{2}$	Variance explained
s(*x*_1_)	5.763	6.696	<0.01	0.689	86%
s(*x*_2_)	7.407	4.542	<0.01		
s(*x*_3_)	8.699	3.391	0.015		

Here, s represents the smooth function, *x*_1_ represents the abundance of the third generation of cotton bollworms of the last generation of the preceding year, *x*_2_ represents minimum winter temperature, and *x*_3_ represents the number of days with the daily minimum air temperature ≤−12°C in winter.

**Figure 6:**
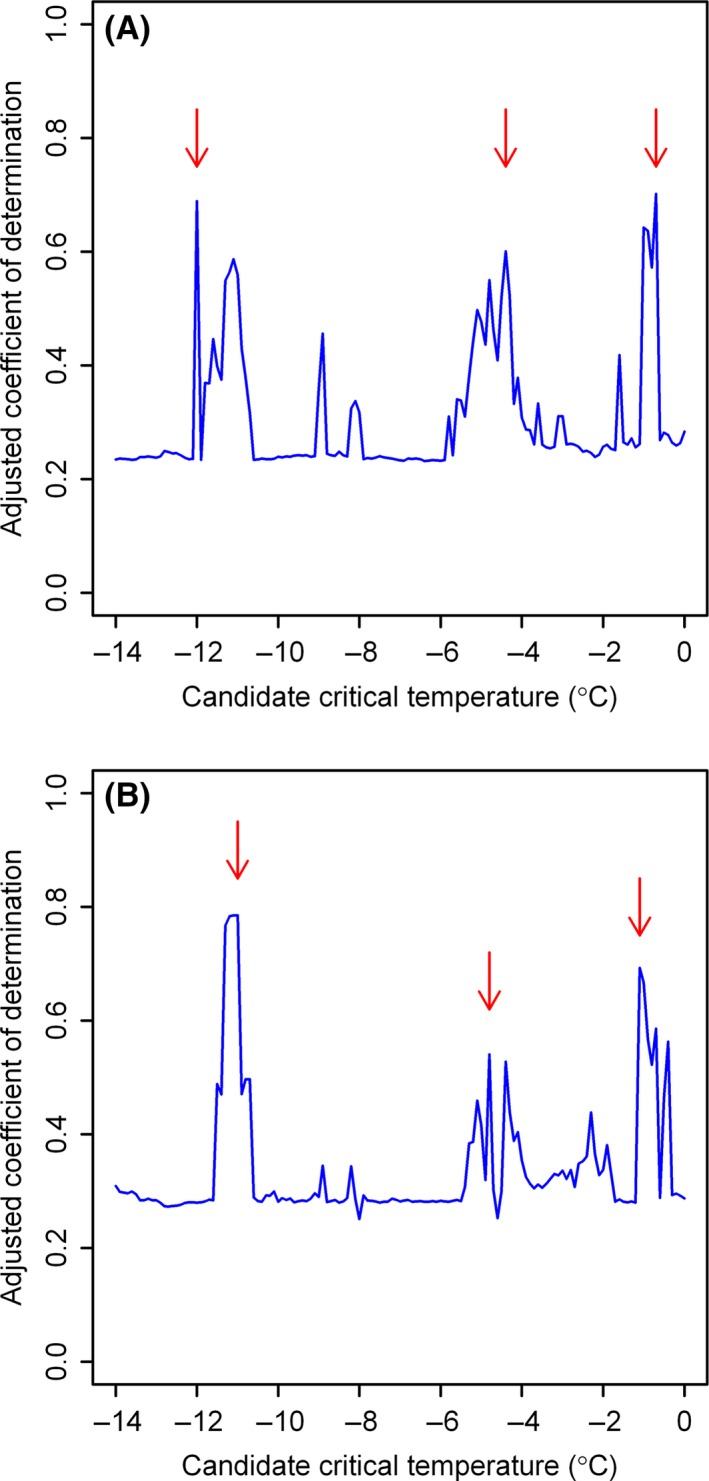
Adjusted coefficients of determination, for *Helicoverpa armigera*, calculated on different candidate critical temperatures, using (A) the minimum winter temperature or (B) winter average temperature, as a predictive variable.

There were three local maximum values for the candidate critical temperature level, −12, −4.4, and −0.7°C (Fig. 6A). However, because we were mainly concerned with the effects of extreme winter temperatures on the mortality of diapausing pupae, the number of days with the minimum air temperature below −10°C was more reliable. Even when we tested the other two candidate critical temperatures, the seesaw effects of MWTs and the number of winter days below the critical temperature were still apparent (see Fig. S1 in the Online Supporting Information).

When the MWT was replaced by the average winter temperature, the goodness of fit from the GAM decreased from 0.689 to 0.280. However, bollworm performance was also sensitive to the choice of critical temperature (Fig. 6B). Using average winter temperature as the predictor with a critical temperature of −11°C, we obtained the highest goodness of fit (Table 3; Fig. 6B). The seesaw effects on bollworm phenology of average winter temperature and the number of days ≤−12°C were still apparent (see Fig. S2 in the Online Supporting Information). Consequently, the critical temperature is between −11 and −12°C, and both the winter average temperature and MWT can be used effectively in the model.

**Table 3:** Generalized additive model fit to the abundance of the overwintering generation of *Helicoverpa armigera*, using three predictive variables, including winter average temperature

Item	df	*F*	*P*	$R_{\mathrm{adj}}^{2}$	Variance explained
s(*x*_1_)	8.907	9.036	<0.01	0.785	92%
s(*x*_4_)	7.413	3.355	0.0244		
s(*x*_5_)	8.099	5.056	0.0035		

Here, s represents the smooth function, *x*_1_ represents the abundance of the third‐generation moth of the preceding year, *x*_4_ represents winter average temperature, and *x*_5_ represents the number of days with the lowest daily air temperature ≤−11°C in winter.

## Discussion

The seesaw effect of match‐mismatch phenology was first proposed in studies on migratory marine fish (Cushing 1969; Anderson et al. 2013). Dietary changes from optimal or adaptive foraging have been found to mitigate the mismatch between breeding phenology and food supply (Zhang and Hui 2014; Nuwagaba et al. 2015). Burger et al. (2012) presented data on the nestling diets of nine populations of pied flycatchers (*Ficedula hypoleuca*) across their breeding range and found that this bird species could adjust its breeding phenology to local climates, with the adjustment sometimes being inefficient due to changes in the phenology of the bird's primary prey (caterpillars). Different components of a food chain cannot be expected to shift their phenology at the same rate and thus are unlikely to remain synchronized in response to climate change (Durant et al. 2007), an expectation consistent with our hypothesis of the mismatch between wheat flowering and overwintering pupal eclosion.

Trophic mismatches are assumed to be greater in migratory species than in sympatric species because of the decoupling of the cues that initiate migration from the state of the target habitat (Anderson et al. 2013). However, here the mismatch can also occur between sympatric species (e.g., cotton bollworm and wheat flowering). In our view, the asynchrony between flowering and eclosion might be caused by differences in effective accumulated temperatures limited by the winter air temperature. The peak date of cotton bollworm pupal eclosion comes earlier compared to the timing of wheat flowering under climate warming. Due to a lack of historical phenological data, we used an indirect approach in this study to confirm the match‐mismatch seesaw effect.

Our results show that the MWT in the current climate is approximate to the optimal temperature for matched phenology of cotton bollworm pupal eclosion and wheat flowering (Figs. 4 and 5B), suggesting that this pest will erupt to high densities more frequently in the coming decades. However, Wu et al. (2008) reported a trend of declining pest outbreaks due to the wide use of transgenic cotton in northern China. They considered that it was caused by the wide plantation of Bt cotton in northern China. However, in China, the Bt cotton is only from a single Bt toxin strain and cotton bollworms are easy to develop the resistance to such a single toxin. A better strategy is to use other transgenic cotton with multiple Bt toxin combination (Shi et al. 2015). However, even though using the effective bi‐toxin cotton, it is still impossible to kill all cotton bollworms in the field. The population dynamics of any species is usually the interaction results between biological phenomena and environment factors, especially climatic factors. Thus, we state that the transgenic cotton should be not the exclusive factor used from explaining the declining outbreaks of cotton bollworms. It is also possible that the decline of cotton bollworms might be caused by the reduction of cotton planting in the Huaihe River Basin of mid‐eastern China, where the MAT ranges from −15 to −10°C (Shi 2013). This range of MATs is sufficient to sustain the diapausing pupae during winter. Declining cotton production in this region is mainly due to reduced labor availability over the past 30 years (Wang 2014); it is unlikely that large numbers of farmers will return in this region (Zhao 2008). The cotton producing industry has struggled to obtain economic returns comparable to the income from other industries, especially construction in southern China, resulting in a serious reduction in the agriculture in the Huaihe River Basin. This has in turn reduced the available hosts for the cotton bollworm. This study suggests that outbreaks of cotton bollworms in northern China may be further suppressed by warmer winter temperatures combined with a reduction of cotton planting in mid‐eastern China.

This study provides new insight into the influence of climate change on the population dynamics of insect pests. Previously, it has been assumed that the population density of insects at mid‐ and high latitudes would increase in response to global warming (Deutsch et al. 2008). It has been assumed that increasing winter temperatures would reduce overwintering mortality of many agricultural and forest insect pests (Ungerer et al. 1999; Kiritani 2007) and that increasing average temperatures throughout the periods of insect growth and reproduction would enhance developmental and growth rates and increase the number of generations per year for multivoltine insects (Deutsch et al. 2008; Amarasekare and Savage 2012). Population declines in some insect species (contrary to the above hypotheses) are often attributed to indirect factors such as a change in farming practices (Kiritani 1988), the introduction of transgenic crops (Wu et al. 2008), the effects of night warming on population dynamics (Zhao et al. 2014), and intensification of agricultural management (Ouyang et al. 2014). The effects of mismatched phenology between insects and their host plants on the population dynamics of insects owing to climate change have been largely neglected.

Our study demonstrates that more attention should be paid to the effect of cumulative temperature on the phenological events of insects and their host plants. Considering that the effects of rearing temperatures on the development and growth of many insects have been widely studied (e.g., Kiritani 2011), future investigations should concentrate on the effects of constant rearing temperature and natural variable air temperature on insect development and growth on different stages of crops (Ikemoto and Egami 2013). Base temperature and thermal time (Trudgill and Perry 1994; Voorend et al. 2014) required by plants to complete a certain phenological event should be calculated from laboratory experiments or long‐term phenological records with corresponding microclimate data (Shi et al. 2014). It would then be possible to compare these two thermal constants between insects and their host plants (Shi et al. 2010) to explore whether or not the phenology of key events of interest are synchronized.

The present study also has implications for possible changes in pest community structures under greenhouse conditions. Rising temperatures may have different influences on phenologies of crops and insects. While traditional insect pests might be reduced through mismatched phenology, greenhouse pests like whitefly that have a greater capacity for high temperatures may increase their population densities. Such mismatched phenology also may provide opportunities for new pests to emerge if the phenology of minor pest species better match crop phenology under changed climate conditions. For example, Lu et al. (2010) reported that outbreaks of mirid bugs (Heteroptera: Miridae) in Bt cotton fields have become more frequent, and the species is now a dominant insect pest of the cotton crop. While transgenic cotton can kill the majority of cotton bollworms that are initially sensitive to Bt toxins (Roush 1998; Wei et al. 2014; Shi et al. 2015), the evolution of resistance to Bt toxins in pests will gradually weaken the effects of Bt cotton (Tabashnik 1994). This interaction between transgenic crops and climate change on the population dynamics of insect pests merits further investigation. Further investigation is needed to directly demonstrate the mismatch based on detailed observations of wheat flowering and pupal eclosion times.

## Conflict of Interest

The authors have no conflict of interests to declare.

## Supporting information


**Figure S1.** Generalized additive model predictions of the abundance of the overwintering generation of *Helicoverpa armigera* based on the number of days with the minimum temperature below two different critical temperatures as a predictor.

**Figure S2.** Generalized additive model predictions of the abundance of the overwintering generation of *Helicoverpa armigera* based on winter average temperature as a predictor where (A) is the smooth and partial residuals of winter average temperature and (B) presents smooth and partial residuals of number of days with the lowest daily air temperature ≤−11°C in winter.
