# Supplementary figures and images for: The seesaw effect of winter temperature change on the recruitment of cotton bollworms Helicoverpa armigera through mismatched phenology
Source: Ecol Evol. 2015 Nov 17;5(23):5652–61. doi: 10.1002/ece3.1829 (PMC4813116; doi:10.1002/ece3.1829)

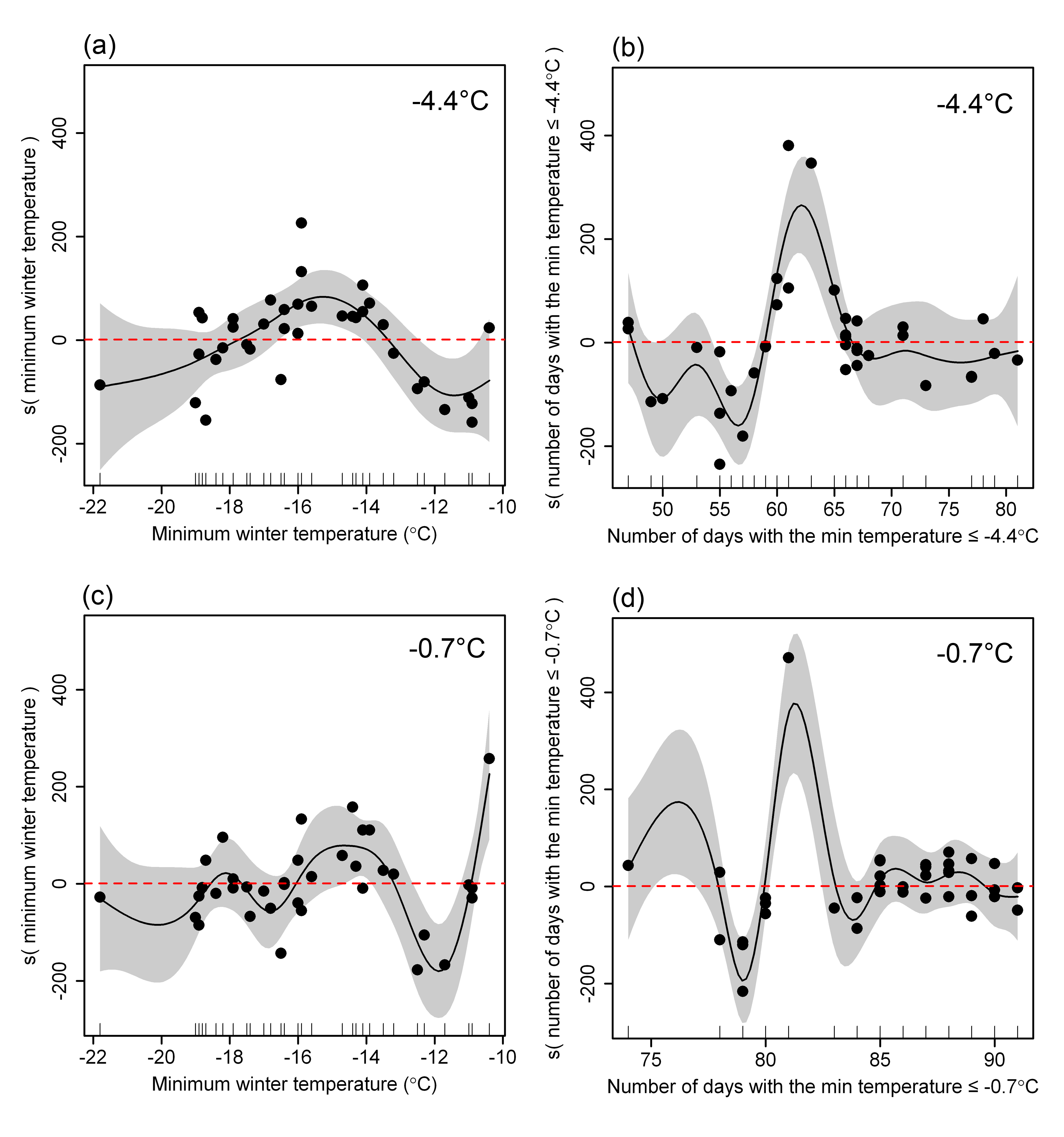

Supplement: Supplementary file 1 — Figure S1. Generalized additive model predictions of the abundance of the overwintering generation of Helicoverpa armigera based on the number of days with the minimum temperature below two different critical temperatures as a predictor. [file ECE3-5-5652-s001.tif]

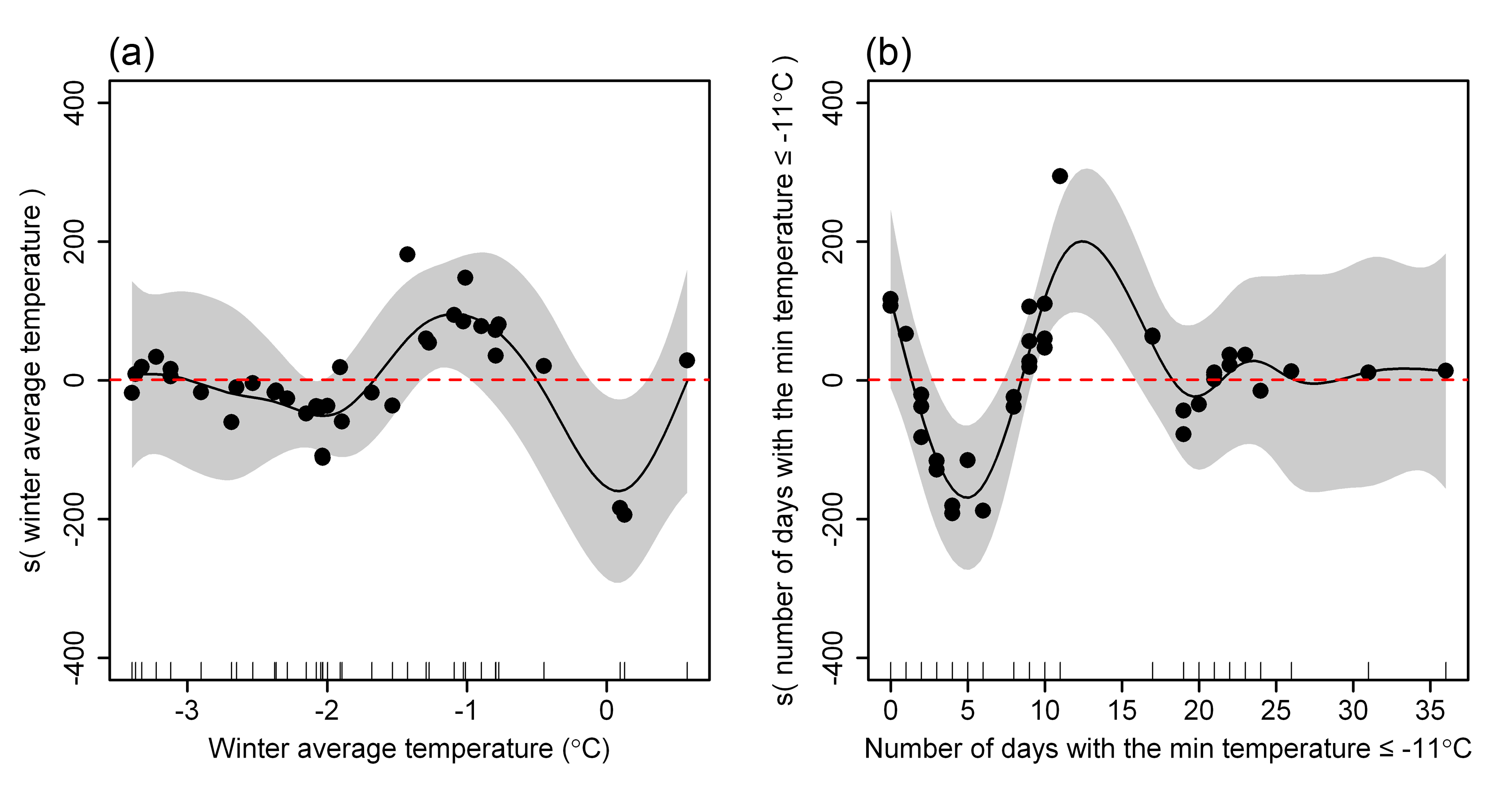

Supplement: Supplementary file 2 — Figure S2. Generalized additive model predictions of the abundance of the overwintering generation of Helicoverpa armigera based on winter average temperature as a predictor where (A) is the smooth and partial residuals of winter average temperature and (B) presents smooth and partial residuals of number of days with the lowest daily air temperature ≤−11°C in winter. [file ECE3-5-5652-s002.tif]
